# *Notes from the Field:* Doubling of Cyclosporiasis Cases Partially Attributable to a Salad Kit — Florida, 2021–2022

**DOI:** 10.15585/mmwr.mm7227a3

**Published:** 2023-07-07

**Authors:** Paul Rehme

**Affiliations:** 1 Florida Department of Health.

Cyclosporiasis is a gastrointestinal infection caused by a protozoan parasite, *Cyclospora cayetanensis*. This species is only known to infect humans and is acquired when oocysts are ingested through food or water contaminated with feces that contain the parasite. The illness was first reported in 1979, and the organism was identified and named in 1994 (*1*). Historically, infections were typically acquired outside of the United States or from produce that was imported into the United States (*1*). In recent years, the number of reported U.S. cases has been increasing: cases more than doubled from 537 in 2016 to 1,194 in 2017, and then nearly tripled, to 3,519 cases in 2018; in 2019, 4,703 cyclosporiasis cases were reported.* Recently, the parasite has been found on domestically grown produce (*2*), and infections have been attributed to these foods (*3*). Produce washing will decrease but not eliminate the parasite (*1*).

## Investigation and Outcomes

In Florida, reported numbers of cyclosporiasis cases have been increasing over the last 10 years†; 254 cases were reported in Florida in 2021, and the number doubled to 513 in 2022, including 486 (95%) laboratory-confirmed cases and 27 (5%) probable cases. Specimens from 276 (54%) cyclosporiasis patients were submitted to CDC’s *Cyclospora* genotyping project, including 211 (76%) which were matched to a specific temporal-genetic cluster code§ (*4*). Among the 513 cases reported in 2022, 469 (91%) patients reported illness onset during May 1–August 31, 2022, with a peak in early July (Figure).

**FIGURE:**
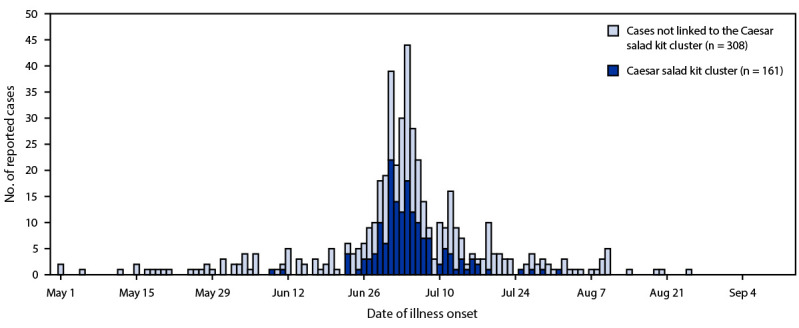
Cyclosporiasis cases (N = 469) — Florida, May 1–August 31, 2022 <Fig_Medium></Fig_Medium>

The Florida Department of Health required that county public health personnel complete the CDC Cyclosporiasis National Hypothesis Generating Questionnaire (CNHGQ)¶ for all patients with illness onset dates during May 1–August 31, 2022. Among 457 completed questionnaires 330 (72%) respondents reported exposure information with no international travel, including 200 (61%) who reported exposure to bagged salad, a commercially produced package of prewashed salad greens. Among respondents reporting exposure to bagged salad, 85 (43%) noted a specific brand of Caesar salad kit containing only romaine lettuce, from a specific grocery store chain. Onset dates for this case cluster occurred during June 23–July 16, with a median disease onset date of July 1. An additional 76 persons with cyclosporiasis reported exposure to Caesar salad kits, but these persons either could not recall the salad kit brands or had purchased them from a different chain for a total of 161 potentially linked cases. Outbreaks of cyclosporiasis have been previously linked to bagged salads in the past (*4*). This activity was reviewed by CDC and was conducted consistent with applicable federal law and CDC policy.**

CDC uses a genotyping tool to aid epidemiologic case linkage in near-real time. Among 211 successfully genotyped specimens from Florida, 153 (73%) were assigned to the same temporal genetic cluster (2022_001), including 43 (96%) of 45 genotyped specimens linked to the bagged salad cluster and 30 (39%) of the 76 persons reporting Caesar salad kits with no further identifying information. This information was shared with the Food and Drug Administration along with source information for the implicated product from the grocery store chain to facilitate traceback of the product; however, the source of the likely contaminated product was not identified.

## Preliminary Conclusions

In this investigation, results from genotyping analysis demonstrated strong agreement between the genotyping and epidemiologic data. The combination of the completed CNHGQ and genetic data strengthens evidence for identifying cases potentially linked to the same source of infection and can guide future investigations.

## Footnotes

* https://wonder.cdc.gov/nndss-annual-summary.html

†
https://www.flhealthcharts.gov/charts/CommunicableDiseases/default.aspx

§ Collection of genetically linked specimens within the same 2-week period were initially assigned temporal-genetic cluster codes, after which a sliding window was applied such that the earliest and latest collection dates could be adjusted to account for specimens that were not collected within the initial 2-week period assigned to the temporal-genetic cluster, but which were genetically linked to the cluster. This process is continued until genetically linked specimens are not detected 7 days before or after the latest dates and allows for the time required for patients to submit specimens after illness onset.

¶
https://www.cdc.gov/parasites/cyclosporiasis/resources/pdf/CNHGQ_2021.pdf

** 45 C.F.R. part 46.102(I)(2), 21 C.F.R. part 56; 42 U.S.C. Sect. 241 (d); 5 U.S.C. Sect. 552a; 44 U.S.C. Sect. 3501 et seq
